# Recent Advances and Applications of Textile Technology in Patient Monitoring

**DOI:** 10.3390/healthcare11233066

**Published:** 2023-11-29

**Authors:** Lindsay Stern, Atena Roshan Fekr

**Affiliations:** 1 Institute of Biomedical Engineering, University of Toronto, Toronto, ON M5S 3G9, Canada; atena.roshanfekr@uhn.ca; 2 KITE Research Institute, Toronto Rehabilitation Institute, University Health Network, Toronto, ON M5G 2C4, Canada

**Keywords:** pressure injury, sleep quality, sleep monitoring, body position monitoring

## Abstract

Sleep monitoring has become a prevalent area of research where body position and physiological data, such as heart rate and respiratory rate, are monitored. Numerous critical health problems are associated with poor sleep, such as pressure sore development, sleep disorders, and low sleep quality, which can lead to an increased risk of falls, cardiovascular diseases, and obesity. Current monitoring systems can be costly, laborious, and taxing on hospital resources. This paper reviews the most recent solutions for contactless textile technology in the form of bed sheets or mats to monitor body positions, vital signs, and sleep, both commercially and in the literature. This paper is organized into four categories: body position and movement monitoring, physiological monitoring, sleep monitoring, and commercial products. A detailed performance evaluation was carried out, considering the detection accuracy as well as the sensor types and algorithms used. The areas that need further research and the challenges for each category are discussed in detail.

## 1. Introduction

Sleep monitoring has become an area of interest to many researchers and BioTech companies. Sleep monitoring research and commercial products primarily focus on monitoring body positions and movements as well as physiological signals to evaluate pressure sores, sleep disorders, and sleep quality.

Pressure monitoring during sleep is an important factor which helps to minimize the risk of pressure sore development. Pressure sores can develop when there is a constant pressure applied to a specific region on the body, resulting in injury to the surrounding skin or tissue region due to cell death, inflammation, and ischemia [1]. In 2020, pressure-related injury complications were responsible for the deaths of roughly 60,000 people worldwide [2]. Patients with pressure sores are 4.5 times more likely to die than those who have similar health factors with no pressure-related injuries [2]. According to the Canadian Association of Wound Care, 70% of pressure related injuries in healthcare settings are preventable [3]. Currently, pressure sores are monitored by healthcare workers in hospitals who are responsible for shifting the body positions of patients throughout the day and night. This can be a laborious task for these workers and can be taxing on hospital resources [4]. According to the Canadian Association of Wound Care, where pressure injury is the main cause for admission, the mean cost of hospitalization is CAD 23,922 ± 54,367 [3]. Therefore, an unobtrusive monitoring technique is necessary to assist patients in and outside of the hospital while reducing the workload of their caregivers.

Previous research showed that body postures and movement during sleep are closely associated with sleep quality [5,6,7]. Good sleep quality is beneficial as it can improve work efficiency, strengthen the immune system, and help maintain one’s physical health [8]. On the other hand, poor sleep quality can cause extreme fatigue and emotional exhaustion and increase the risk of cardiovascular diseases, as well as obesity and diabetes [8,9]. An unobtrusive monitoring system could assist people with understanding their quality of sleep.

Previous studies also demonstrated that position monitoring of the elderly population plays an important role in fall prevention and sleep disorder diagnosis [10,11,12,13]. Falls can often occur when a person attempts to get out of bed or becomes close to the edge of the bed during sleep. Falling from the bed causes serious injuries, such as bruises, achiness, and bone fracture [10]. In addition, remaining in the same sleep posture for a long time will not only result in pressure sores but also other health issues, such as sleep paralysis and nocturnal gastroesophageal reflux [11]. Therefore, an unobtrusive posture monitoring system is critical for the elderly population, who are more susceptible to injuries and health complications.

Monitoring physiological data during sleep such as the respiratory rate and heart rate can help in daily health assessment. Such monitoring can also aid in the early detection of issues like cardiovascular diseases, obstructive sleep apnea (OSA), and mental stress [14,15]. According to the Canadian Chronic Disease Surveillance System, as of 2018, 2.6 million Canadian adults over the age of 20 live with a diagnosed heart disease [16]. Adults 40 years and over with diagnosed heart failure are 6.3 times more at risk of death if not monitored properly [16]. Respiratory monitoring is also important, as 12.8% of the elderly population (60+ years old) reported being diagnosed with OSA, where males are 4.8% more likely to have OSA than their female counterparts [17]. OSA can be defined as the inability to get enough air during sleep, lowering oxygen blood levels [18]. This pattern may be repeated on average between 5 and 30 times per hour, impairing one’s ability to reach a deep sleep state [18,19]. Inadequate restful sleep may cause cardiac diseases such as hypertension, stroke, and heart failure. Severe OSA can also be the reason for decreased memory and cognitive decline [20]. However, 80% of people with symptoms of OSA go undiagnosed due to costly and time-consuming diagnostic procedures [19]. Although the polysomnography (PSG) is known as the gold standard in sleep disorder diagnosis, it is complicated, expensive, time-consuming, and must be used inside a laboratory. Furthermore, it is difficult for people to receive this diagnosis as there are few hospitals which provide PSG tests, especially in rural areas. Therefore, there is a demand for a cost-effective, easy-to-set-up tool with acceptable accuracy to monitor physiological data during sleep and detect sleep disorders.

To address these challenges, several studies worked on contactless textile technology in the form of bed sheets or mats to monitor body positions, vital signs, and sleep. In this paper, we review and compare both the prior literature and commercial devices related to this technology, discussing the field’s advancements as well as the existing gaps.

This paper is organized as follows. Section 2 discusses the process of how this literature search was conducted as well as the exclusion criteria. Section 3 explains the recent literature and commercial products within the field of smart bed sheets and mats for in-bed posture monitoring, physiological signal monitoring, and sleep monitoring. Section 4 discusses the pros and cons of the research and existing products explained in Section 3 in more detail. Finally, Section 5 concludes the review and discusses the next steps that should be taken within this field of research.

## 2. Methods

The literature search was conducted from April to July 2023. Google Scholar, Engineering Village, and the University of Toronto’s Online Library Database were the search engines used for this review paper. Databases such as IEEExplorer, PubMed, and MDPI were also searched for papers. The following is a list of the key search terms used, where a “+” signifies “and” and a “/” signifies “or”: smart mat/sheet, sleep mat/sheet, smart mat/sheet + body posture/position, smart mat/sheet + body movement, sleep mat/sheet + body posture/position, sleep mat/sheet + body movement, smart mat/sheet + physiological data, sleep mat/sheet + physiological data, smart mat/sheet + respiratory/breathing rate, sleep mat/sheet + respiratory/breathing rate, smart mat/sheet + heart rate/heartbeat, sleep mat/sheet + heart rate/heartbeat, smart mat/sheet + sleep apnea monitoring/detection/diagnosis, sleep mat/sheet + sleep apnea monitoring/detection/diagnosis, smart mat/sheet + sleep quality/sleep cycle/sleep stage, and sleep mat/sheet + sleep quality/sleep cycle/sleep stage.

In total, 95 papers were analyzed, and 63 relevant papers were selected for this review. Since the review was focused on the use of smart mats and sheets or sleep mats and sheets, any studies or commercial products that incorporated a bed mattress were removed. Papers and commercial products that focused on smart mats and sheets or sleep mats and sheets that only examined the body or room temperature were also removed, as these areas were not the focus of this study. As this is a state-of-the-art review paper, we focused on papers from the past 13 years (2010–2023).

## 3. Applications of Textile Technologies for In-Bed Patient Monitoring

This section is composed of four subsections. The first subsection discusses the existing literature on the application of smart bed sheets and mats in detecting and classifying in-bed postures using machine learning algorithms. The subsequent subsection reviews previous studies on detecting the respiratory rate and heart rate from data collected by smart bed sheets or mats. The third subsection investigates the accuracy within the literature with regard to detecting obstructive sleep apnea as well as sleep quality using data collected by smart bed sheets and mats. Finally, the last subsection summarizes the smart mats or bed sheets on the market that are used to detect in-bed body positions, in-bed body movement, or in-bed vital signs.

### 3.1. Body Position and Movement Monitoring Using a Smart Mat or Bedsheet

Body position monitoring has been an area of research over the past couple of decades. This is an area of interest as sleep postures can contribute to sleep disorders, pressure ulcers, and pain in the shoulder, neck, and back regions [8]. In recent studies, sleep posture and body movement monitoring has been applied to determine sleep quality and sleep efficiency as well as warn caregivers whether a person is near the edge of the bed [8,10]. Today, monitoring body positions during sleep typically includes the use of either a video infrared camera or wearable devices. However, there are concerns with the use of these systems. Video infrared cameras are sensitive to environmental changes such as blanket movement and suffer from occlusion issues. Wearable devices such as watches [21] and chest straps [22] tend to be obtrusive to sleep, reducing a person’s sleep quality, and are sensitive to motion artifacts [8]. Therefore, a variety of sleep monitoring mats have been studied which create an unobtrusive monitoring system for detecting body position and motion.

In this section, 29 articles have been reviewed with a focus on body position and movement detection. All these studies were conducted in either a simulated or clinical testing environment. A simulated testing environment typically relates to the supervisor instructing the subject to lie in specific postures for a short period of time (less than 1 h), which can be seen in [23,24,25,26,27,28,29,30]. Contrastingly, a clinical testing environment allows the subject to alter their body position for their comfort for a minimum of 1 h. However, typically this study is conducted overnight for a minimum of 7 h, which can be seen in [31,32,33,34]. Diao et al. presented the outcomes from both clinical and simulated testing environments for detecting body postures in [8,32] using various algorithms. The results showed that the detection rate in a simulated environment had an accuracy of 95.08% and 95.43% in [8,32] respectively, while in the clinical testing environment, the detection accuracy dropped to 86.35% and 86.80% in [8,32], respectively. Therefore, as expected, a simulated study can have a higher detection accuracy as it is more controlled. However, sleep is not typically controlled, and therefore a simulated testing environment may not represent actual overnight sleep.

A variety of embedded sensors have been studied to determine the accuracy of detecting sleep positions and movement. These sensors are classified into two main categories: pressure sensors and force sensors. Pressure sensors have been used in many articles, including studies completed by Stern et al. [35], Davoodnia et al. [36], Enokibori et al. [31], and Matar et al. [37]. Force sensors such as force-sensitive resistors (FSRs), resistive sensors, piezoresistive force-based sensors (FSAs), and load cells were used in studies completed by Huang et al. [38], Kitzig et al. [39], and Liu et al. [34]. Although these two categories of sensors use different mechanisms for detecting body position and movement on the bed, they provided similar results in terms of detection accuracy.

The most common machine learning algorithms to use within body posture detection analysis are deep neural networks (NNs), K-nearest neighbor (KNN), and support vector machines (SVMs). Neural networks can be described as a system that has many interconnected nodes and works similar to the neurons of the brain to classify different events and objects [31]. A KNN model is a machine learning algorithm that uses the distance and proximity to classify data into groups [24]. An SVM model is a classifier that uses labeled datasets to train itself to perform classifications or regression analysis on datasets [37].

**Figure 1:**
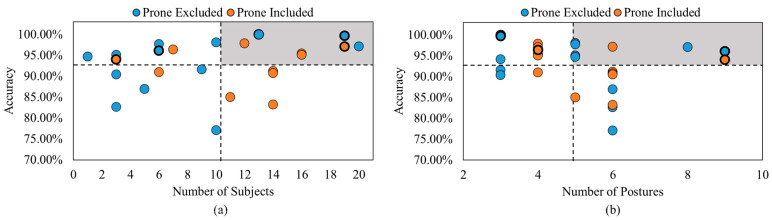
Accuracy comparison between studies that included the prone position and studies that either exclude the prone position or merge it with the supine position (**a**) versus the number of subjects and (**b**) versus the number of postures detected [4,8,10,11,23,24,25,26,27,28,29,30,31,32,33,34,35,36,37,38,40,41,42,43,44,45,46,47].

Several studies ignored classifying supine and prone body positions separately or ignored the prone position entirely. It was determined that it can be difficult to distinguish between these two postures via force and pressure imaging as these two positions have quite similar pressure spots. Indeed, the main difference is that the supine position occurs while one is lying on his or her back, whereas the prone position occurs while lying on the stomach [4]. Figure 1a depicts the detection accuracy of the reviewed articles versus the number of subjects for the posture classification algorithms, and Figure 1b shows the detection accuracy versus the number of postures classified within the algorithm. Both figures consider studies that merge supine and prone positions (orange dots) or exclude the prone position (blue dots). The black dashed lines represent the average accuracy and sample size or positions considering all 20 studies. The gray region shows the region of interest, which has large sample size and high accuracy values in Figure 1a or a high quantity of positions in Figure 1b.

As highlighted in Figure 1a,b, the study in [40] provided a maximum accuracy of 99.97% with 13 subjects and 3 body postures. The study in [31] examined cases where the prone position was included and excluded, resulting in accuracies of 97.1% and 99.7%, respectively. This demonstrated that there was higher accuracy detection when the prone position was excluded. However, the 2.6% difference may not justify excluding this body position, since this position is a part of the four most common sleeping postures. Based on Figure 1a, this study included 19 participants, and from Figure 1b, this study classified 3 and 4 body positions: supine, left side, right side, and prone (as the fourth position). Based on Figure 1b, the studies in [38,41] classified the highest number of body postures, totaling 9 different positions with accuracy rates of 94.05% and 96.10% with and without the prone position, respectively. However, these studies tested three and six participants, as shown in Figure 1a. Among the articles reviewed, the maximum sample size was 300 participants in [42]. This article was excluded from Figure 1 for better visualization. The studies in [11,37] were also excluded from Figure 1a, as these studies did not mention the number of subjects tested. Viriyavit et al. [10] and Tang et al. [11] proposed body position detection for the elderly population of 60+ years old. Both studies examined fall prevention methods for the elderly population when getting off the bed or coming close to the edge of the bed while sleeping. Viriyavit et al. [10] classified an unoccupied bed, sitting, lying in the center, right edge lying, and left edge lying using a combined naïve Bayes and Bayesian network algorithm, with detection accuracies of 98.46%, 89.07%, 96.40%, 98.46%, and 93.05%, respectively. Tang et al. [11] classified an unoccupied bed and a singular posture representing edge detection using a convolution neural network (CNN), with accuracy rates of 100% and 95%, respectively. This study also used a mobile application to detect the postures throughout the night and notify a caregiver of a fall event when edge detection occurred [11].

Table 1 and Table 2 summarize the literature reviewed with merged supine and prone positions or the prone position excluded and the literature that included both the prone and supine positions, respectively.

### 3.2. Physiological Data Monitoring Using a Smart Mat or Bedsheet

Monitoring physiological data during sleep such as the respiratory rate and heart rate can help monitor daily health and discover abnormalities such as cardiovascular diseases, sleep apnea, and mental stress in the early stages [14,15]. Conventionally, a polysomnography (PSG) monitors physiological data during sleep. However, this device requires subjects to be in sleep laboratories and attach several sensors to their bodies during sleep. Although PSG is the “gold standard” for monitoring physiological data during sleep, it can be obstructive to normal sleep, and the sensors may fall off the body, causing inaccurate results [14]. Wearable devices such as smart watches and chest straps [48,49] have become increasingly popular to monitor physiological data. However, these devices must be in direct contact with the skin and are susceptible to motion artifacts [14]. Contactless smart mats or cover sheets are viable solutions for establishing an unobtrusive monitoring system for physiological data in laying down positions.

In this section, 15 academic papers are reviewed relating to detection of the respiratory rate and heart rate using a smart bedsheet or mat. Similar to the body posture detection section, clinical and simulated testing environments were used to evaluate the smart mats and bed sheets with respect to respiratory rate and heart rate. Seven out of 15 studies only used a simulated setting [14,50,51,52,53,54,55], while the rest used the clinical testing environment, allowing for more reliable results. Only [15] used both clinical and simulated testing environments, with a simulated environment only having 0.9% higher heart rate coverage than a clinical environment, demonstrating that these studies should also be evaluated in a clinical setting to show the potential use in a realistic situation. Additionally, only two sensor categories were studied to determine the accuracy of detecting physiological data: pressure sensors and force sensors. There was no significant discrepancy in accuracy between these two methods when it came to detecting physiological data. A variety of algorithms were used for detecting the respiratory rate and heart rate. The most common detection method was peak detection, which identifies the peaks of the signal to determine each breath or beat [15,50,56,57,58]. Maximal overlap discrete wavelet transform (MODWT), wavelet analysis, cepstrum, clustering, and empirical mode of decomposition (EMD) are also popular detection algorithms for detecting the signals of the heart rate and respiratory rate, which can be seen in [12,14,51,59]. Peng et al. [14] compared the wavelet analysis and EMD for estimating the respiratory rate and heart rate. This study found that wavelet analysis was more accurate than the EMD method by 3.83% for detecting the respiratory rate and by 1.92% for detecting the heart rate. This study also considered different window sizes (10 s, 30 s, and 60 s) using three algorithms: wavelet analysis, EMD, and dynamic smoothing (DS). Their findings indicated that increased window sizes consistently yielded more accurate results.

Huang et al. in [53] used a de-shape synchrosqueezing transform (DSST) to evaluate the respiratory rate and heart rate and consider both the time and frequency domains’ features. The authors decided to use two methods when evaluating the respiratory rate: examining the shoulder region and examining the torso-weighted centroid. The DSST was performed on filtered data obtained from nonzero values found in the shoulder blade regions, resulting in a root mean squared error (RMSE) of 1.32 bpm. The DSST was then performed on filtered data obtained from the vertical movement of the weighted centroid in the torso region, which mimics inhalation and exhalation, resulting in an RMSE of 0.87 bpm. Therefore, the analysis of data from the torso-weighted centroid resulted in a more accurate respiration rate. When employing the DSST for heart rate estimation, an RMSE of 5.55 bpm was obtained, which was significantly higher than the results achieved for respiratory rate estimation. This suggests that [53] demonstrated superior performance in detecting the respiratory rate compared with heart rate detection.

The health of the participants in each study can influence the accuracy of the outcomes. Those with histories of cardiac or respiratory illnesses or sleep disorders may skew the accuracy, often resulting in lower results compared with studies with healthy subjects. The study completed by Kortelainen et al. [60] included subjects with sleep problems and arrhythmias. The authors found that when considering only the healthy subjects, the heart rate’s mean absolute error (MAE) was 0.4% with coverage of 88%. However, including unhealthy subjects increased the MAE to 1.8% with coverage of 80%. This might indicate that studies that include either healthy and unhealthy subjects or just unhealthy subjects are susceptible to lower accuracy rates.

Seven of the 15 articles estimated both the respiratory rate and heart rate [12,14,50,53,56,60,61], but only [50] reported the results in a consistent format for comparison. From this, the MAE relating to the respiratory rate was slightly lower than that of the heart rate, indicating that it may be easier to correctly detect the respiratory rate from smart mats or bedsheets than the heart rate.

**Figure 2:**
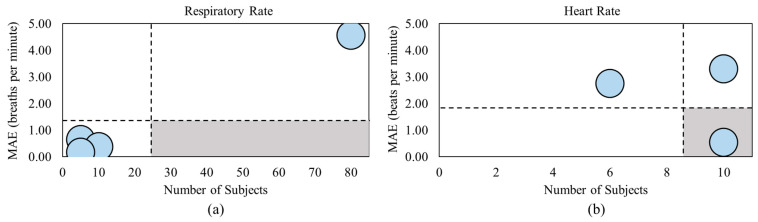
MAE comparison between studies that captured (**a**) respiratory rate and (**b**) heart rate [50,51,54,55,58,59].

Figure 2a highlights the MAEs of four out of the nine articles on respiratory rate estimation using smart mats or bedsheets [50,55,58,59]. Similarly, 3 of the 12 heart rate articles investigated the MAEs of their smart mats or bed sheets [50,51,54], shown in Figure 2b. The black dashed lines represent the average MAE and number of subjects of all included studies. It should be noted that the vertical black dashed line in Figure 2a is skewed to the higher end of the number of subjects due to the large sample size in [59]. The gray region shows the region of interest, which has a large sample size and low MAE values.

The minimum MAE relating to the respiratory rate was from [58], with an MAE value of 0.18 breaths per minute. However, this article also included the smallest sample size of only five subjects. Though [50] did not fall within the gray region of Figure 2a, it was the closest study to this region due to the low MAE value of 0.38 breaths per minute and the second highest sample size of 10 subjects. Meanwhile, [59] consisted of the largest sample size with 80 subjects but also achieved the highest MAE of 4.57 breaths per minute. The minimum MAE relating to the heart rate was achieved in [50], with an MAE value of 0.55 beats per minute and the largest sample size of 10 subjects, falling within the gray region of Figure 2b. By comparison, the study in [54] achieved an MAE over six times greater than that in [50] with the same sample size. Table 3 and Table 4 summarize the articles reviewed in this section.

### 3.3. Sleep Monitoring Using a Smart Mat or Bedsheet

Combining the body posture and movement detection as well as physiological data can assist with diagnosing sleep apnea and determining the sleep quality of a person.

Sleep apnea is a sleep condition that affects many individuals and is related to health complications. PSG is the gold standard for assessing and diagnosing sleep apnea [62]. PSG uses breathing sensors to detect nasal-oral airflow amplitude and blood oxygen saturation (SaO_2_) levels [63]. PSG also monitors electroencephalogram (EEG) signals to measure brain activity, electrooculogram (EOG) signals to measure eye movement, electromyography (EMG) signals to measure muscle activity, and electrocardiogram (ECG) signals to measure the heart rate [64]. This test must occur overnight and can be intrusive and disruptive to the patient’s sleep [62]. Additionally, PSG data require manual scoring of apneic events and thus can be time-consuming and require special training [63]. PSG devices are also expensive and cannot be used in a person’s home [20]. Therefore, a smart mat that can detect and diagnose sleep apnea is a viable alternative that makes this process more affordable and accessible to all.

The quality of sleep, rather than the quantity, is associated with health [13]. Therefore, sleep quality analysis is becoming important to study and being used to identify a variety of health challenges. According to The American Academy of Sleep Medicine (AASM), a typical sleep cycle consists of non-rapid eye movement (REM) sleep, meaning deep sleep, and REM sleep, meaning light sleep [13]. This pattern repeats cyclically throughout the night. In healthy adults, an average sleep cycle lasts between 90 and 100 min, beginning with three stages of non-REM sleep followed by REM sleep. REM sleep comprises about 20–25% of total sleep in typical healthy adults. Generally, the longer the non-REM sleep episode, the better the quality of sleep [13]. This cyclic pattern of the non-REM and REM stages is altered for subjects with sleep disorders and other illnesses due to wakefulness throughout the night [13]. Therefore, sleep cycle analysis can generate information about people’s sleep quality, specifically those suffering from sleep disorders.

We reviewed six academic papers related to sleep apnea and five concerning sleep quality. Almost all studies were conducted in a clinical environment to better detect apneic and sleep cycle events. These studies used either pressure or force sensors, similar to body posture and physiological data detection. They all used machine learning algorithms and peak detection since the data included body movement, the respiratory rate, and the heart rate.

The study conducted by Wang et al. [20] included the maximum number of subjects (136) compared with other studies. They tested 41 subjects with no diagnosis of sleep apnea, 23 subjects with mild sleep apnea, 34 subjects with moderate sleep apnea, and 38 subjects with severe sleep apnea.

It was reported that the sleep mat resulted in 95.1%, 91.3%, 94.1%, and 94.7% detection accuracy values for no, mild, moderate, and severe sleep apnea, respectively, using an SVM machine learning model. The study conducted by Hwang et al. [63] consisted of 4 subjects with no diagnosis of sleep apnea, 10 subjects with mild sleep apnea, 7 subjects with moderate sleep apnea, and 10 subjects with severe sleep apnea. Through conducting the same study on all these subjects overnight, it was found that the sleep mat resulted in 95.4%, 88.4%, 82.9%, and 80% accuracy detection for detecting no, mild, moderate, and severe sleep apnea, respectively [63].

The studies in [12,50] only examined the accuracy of detecting apneic events in subjects with OSA. The authors of [12] detected apneic events using derived respiratory signals via a sliding window of 60 s and 30 s, resulting in an accuracy of 49.96% and 54.33%, respectively. The authors of [50] detected apneic events in 10 subjects: 4 with diagnosed moderate sleep apnea and 6 with severe sleep apnea. The obtained sensitivity of 24.24% indicates the correctly identified apneic events, whereas the specificity of 85.88% correlates to the correctly identified non-apneic events [50]. Similarly, the study in [65] detected the possibility of apneic episodes during 3 consecutive nights in 2 healthy subjects and 7 subjects diagnosed with OSA, resulting in an accuracy of 71.9%.

The study conducted by Jung et al. [66] evaluated the sleep quality of 20 subjects: 10 subjects with no medical concerns or sleep disorders and 10 with OSA. The nighttime wakefulness (awakening after sleep onset) was estimated by a polyvinylidene fluoride (PVDF) film sensor with an accuracy of 97.4% in the healthy subjects and 96.5% in the patients with OSA. The sleep efficiency was estimated with 1.08% and 1.44% errors for the healthy subjects and patients with OSA, respectively.

Studies conducted by Samy et al., Laurino et al., and Kortelainen et al. evaluated the detection rates of their systems considering wakefulness, non-REM sleep, and REM sleep [13,67,68]. Samy et al. [13] detected the episodes of wakefulness, non-REM sleep, and REM sleep with 55.9%, 100%, and 38.2% accuracy, respectively. Laurino et al. [67] reported an accuracy of 83% for detection of the wakefulness episodes, 83% for non-REM sleep and 79% for REM sleep. Laurino et al. [67] also examined the possibility of detecting no bed occupancy, which resulted in an accuracy of 99%. Kortelainen et al. [68] reported an accuracy of 81% for detection of the wakefulness episodes, 75% for non-REM sleep, and 80% for REM sleep. Kortelainen et al. [68] also examined sleep efficiency, resulting in an overall detection accuracy of 84%.

A further comparison of sleep apnea detection studies and sleep quality evaluation studies along with their associated algorithms is shown in Table 5 and Table 6, respectively. It is worth mentioning that it was difficult to conclude which signals, sensors, and algorithms are best to use as these characteristics were different between each study. For example, two studies [50,69] utilized the same signals (respiratory rate, heart rate, and body movement), and both included peak detection. However, the study in [69], which involved 96 patients with sleep apnea ranging from none to severe and used piezoelectric sensors, achieved a notably higher sensitivity rate of 88%. In contrast, the study in [50] involved only 10 patients, all with moderate or severe sleep apnea, and used a fiber optic sensor for a 30 min study, resulting in a sensitivity of 24.24%. Therefore, although these two studies captured the same signals and used similar detection algorithms, the variation in the patient population and types of sensors makes it difficult to conclude which method is better. Another example is [20], which obtained the highest performance regarding sleep apnea detection with an accuracy of 97.57% while utilizing a combination of respiratory and heart rate signals from a large number of subjects (136). This method utilized a micro-movement-sensitive mattress and a random forest machine learning algorithm to detect sleep apnea events. In [66], the authors achieved the highest performance regarding sleep quality monitoring by combining heart rate and body movement signals from 20 participants, resulting in an accuracy of 96.95%. They used a polyvinyl fluoride film sensor and a peak detection algorithm. However, more recent research, such as [70], achieved a slightly lower accuracy of 90.9% using data from 25 subjects with only body movement signals and a hidden Markov model but with the advantages of a larger sample size and the use of pressure sensors.

### 3.4. Commercial Smart Mats or Bedsheets

Five commercial products along with associated validation studies have been reviewed to evaluate smart mat products available for purchase that can detect either body posture and movement or physiological signals.

#### 3.4.1. Withings Sleep Tracking Mat

**Figure 3:**
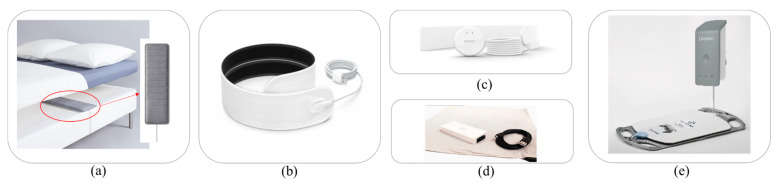
(**a**) Withings Sleep Tracking Mat [71], (**b**) Beddit Sleep Monitoring Mat [73], (**c**) Emfit sleep monitoring mat [74], (**d**) Studio 1 Labs bed sheet [77], and (**e**) EalrySense monitoring system [78].

The Withings Sleep Tracking Mat shown in Figure 3a is owned by Withings with the headquarters located in Issy-les-Moulineaux, France, however, provides services globally. This smart mat is placed underneath the mattress at torso level to analyze the respiratory rate, heart rate, and body movement via pneumatic pressure sensors. It can also detect snoring and the cessation of breathing via sound sensors. Combining these parameters allows this sleep mat to monitor the sleep duration, sleep onset, time to wake, sleep cycle, continuous and average heart rate, and snoring duration. The data collected from the mat can be viewed in an app available on both Android and IOS phones. This sleep mat appears to be a popular choice and costs CAD 127.87 [71].

Two validation studies were conducted on the Withings Sleep Tracking Mat to verify the accuracy in detection of the respiratory rate, heart rate, and sleep apnea [9]. An overnight study was conducted in 2019 on 40 different subjects, where 24 of these subjects (6 healthy and 18 patients) were studied to determine the accuracy of the respiratory rate detection and 37 subjects (6 healthy and 31 patients) were studied to determine the accuracy of the heart rate detection. All data were compared to a PSG machine to validate the accuracy. It was concluded that the Withings Sleep Tracking Mat produced an accuracy of 69.6% and 40% for estimating the respiratory rate and the heart rate, respectively [9]. A recent overnight study was completed in 2021 to determine whether the Withings Sleep Tracking Mat could be used to diagnose sleep apnea in which 118 subjects were studied, ranging from having no sleep apnea to severe sleep apnea. All data were compared to a PSG machine to validate the accuracy. It was determined that the Withings Sleep Tracking Mat can detect subjects with no sleep apnea with an accuracy of 75%, subjects with mild sleep apnea with 47.83% accuracy, subjects with moderate sleep apnea with 51.52% accuracy, and subjects with severe sleep apnea with 86% accuracy [72].

#### 3.4.2. Beddit Sleep Monitoring Mat

The Beddit Sleep Monitoring Mat shown in Figure 3b is an Apple Inc.-owned product, with the headquarters located in San Jose, CA, USA, however, is a global company. This sleep monitoring mat is placed under the mattress to analyze the respiratory rate, heart rate, body movement, and snoring via piezo force sensors, capacitive touch sensors, humidity sensors, temperature sensors, and the microphone of an iPhone. Using these techniques, the sleep mat can monitor the sleep time, bedtime, time to fall asleep, time awake, time away from bed, wake up time, sleep efficiency, snoring time, average, high, and low heart rates, and average breathing time. Additionally, the Beddit mat pairs with an app on iPhones and Apple Watches to visualize the analyzed data. This sleep mat costs CAD 155.46 but does not appear to be available for purchase on the Apple website at this time [73].

One validation study was completed in 2019 to determine the legitimacy of the Beddit Sleep Monitoring Mat with respect to sleep cycle monitoring. This study was completed over 2 non-consecutive nights with 10 young and healthy subjects. All data were compared to a PSG machine to validate the accuracy. It was determined that this mat had 42.1% accuracy for detecting wakefulness, 55.6% accuracy for detecting the REM state, and 37.5% accuracy for detecting the non-REM state. This mat would overestimate the total sleep time by 10.53% and sleep efficiency by 8.73%, and it would underestimate the wakefulness after sleep onset (WASO) by 29.28% [72].

#### 3.4.3. Emfit

The Emfit sleep monitoring mat shown in Figure 3c is owned by Emfit with headquarters located in San Marcos, TX, USA and Vaajakoski, Finland, and serves globally. This sleep monitoring mat is placed under the mattress to analyze the heart rate, respiratory rate, and body movement via patented quazi-piezoelectric sensors. Utilizing these tools, this sleep mat analyzes the sleep time, sleep classes, average, high, and resting heart rate, high, average, and low breathing rate, movement activity (tossing and turning), and bed occupancy and exit. This sleep mat pairs with an app on Android and IOS phones to view the collected data. The Emfit sleep monitoring mat has a subscription service that costs CAD 24.39/month and requires a minimum of a 6 month commitment [74].

Two validation studies were completed on the Emfit sleep monitoring mat that examined the accuracy of the heart rate and respiratory rate detection [74,75]. A 2019 overnight home study was conducted on 20 healthy subjects to determine the accuracy of the heart rate detection of the Emfit sleep monitoring mat. All data were compared to a Firstbeat Bodyguard 2 heart rate monitor. It was concluded that this sleep mat has a root mean square error (RMSE) of 2.4 bpm when detecting the heart rate [75]. A second overnight study was conducted in the same year to validate the accuracy of heart rate and respiratory rate estimation in which 34 subjects were recruited: 8 with obstructive sleep apnea, 9 with prolonged partial obstruction, 6 with periodic limb movement disorder, and 1 with narcolepsy. All data were compared to a PSG machine to validate the accuracy. It was determined that the Emfit sleep monitoring mat had an accuracy of 98.7% for heart rate estimation and 97.6% for respiratory rate estimation [76].

#### 3.4.4. Studio 1 Labs Bed Sheet Monitor

The Studio 1 Labs bed sheet monitoring system is shown in Figure 3d, with headquarters in Markham, ON, Canada, serving the Canadian population. This bed sheet is composed of a pressure-sensing fabric that monitors falls, pressures ulcers, and abnormal breathing patterns. This sheet does not appear to have an associated app but does connect to a computer to view the collected data. Pricing begins at CAD 704.64, but it does not appear to be available for purchase at this time [77].

A single validation study was conducted in 2018 to determine the legitimacy of the Studio 1 Labs bed sheet for respiratory rate estimation. This study was conducted on 21 young and healthy subjects for an 8 h overnight study. All data were compared to a manual counting respiration rate and wrist pulse oximeter to validate the accuracy. It was determined that the Studio 1 Labs bed sheet has an accuracy of 84% when estimating the respiratory rate [19].

#### 3.4.5. EarlySense

The EarlySense monitoring system shown in Figure 3e is owned by Baxter International, with headquarters inRamat Gan, Illinois, USA, however, provides services globally. This monitoring system is placed underneath the mattress and measures the heart rate, respiratory waveforms, and movement via piezoelectric sensors. The EarlySense system is only available for medical use, in long-term care homes, and in rehabilitation centers. Therefore, it cannot be purchased for personal use at home [78].

Two studies were conducted on the EarlySense monitoring system to validate the machine with respect to the heart rate, respiratory rate, and sleep cycle. A 2017 study was conducted overnight using the EarlySense monitoring system on 63 subjects. All data were compared to a PSG machine. It was concluded that the EarlySense system has an accuracy of 96.1% when estimating the heart rate and 93.3% when estimating the respiratory rate [79]. Another overnight validation was conducted on 28 children with suspected sleep-disordered breathing. The EarlySense system provided an 89.1% accuracy for determining whether a subject was asleep, 87.9% accuracy for detecting wakefulness, 79.6% for detecting non-REM sleep, and 48.8% for detecting REM sleep [80]. Table 7 describes a complete summary of all the validation studies completed on the commercial products mentioned above.

## 4. Discussion

This paper reviewed 29 papers about body posture and movement, 15 papers about physiological data monitoring, and 11 papers about sleep apnea and sleep quality detection. Five commercial products were also reviewed with associated validation studies, totaling eight papers.

Sleep monitoring mats that detect body postures and movements have been studied for many years, longer than the time span parameter of this literature review, allowing improvement and for high-accuracy data to be published. Out of the 29 articles that were reviewed, only 7 studies were evaluated in a clinically based testing environment, meaning that these studies were conducted for over 1 h or overnight where the subjects had control over their sleeping positions, simulating a realistic sleeping environment. The 22 other studies were conducted in a simulated setting where the subjects were instructed on which position to lie in with minimal allowance for leg and arm alterations. It is important to note this limitation, as the accuracy of body posture detection of these 22 articles would likely be lowered if they were studied in a clinical environment. Twenty-five of the studies reviewed had a small sample size (<20 subjects), indicating that the accuracy achieved by those studies could have been affected if more subjects were tested.

Sleep monitoring mats that estimate physiological data, such as the respiratory rate and heart rate, are a newer area of study, as previous medical devices such as a PSG system and wearable devices were used. Ten out of these 15 papers only consisted of healthy subjects and subjects with no prior cardiac or respiratory issues or sleep disorders. Four of these studies only included patients with cardiac issues or sleep apnea. Four studies included a mixture of healthy and non-healthy subjects but typically would have healthy subjects as the majority of their subject pool. In these four studies, a distinction was made between the accuracy of physiological parameter estimation for healthy subjects and non-healthy subjects, and it was evident that the results from the non-healthy subjects reduced the average accuracy due to a lower signal-to-noise ratio. Additionally, 13 studies included a small sample (<20), indicating that the accuracy achieved by each study could have been affected if more subjects were evaluated. Finally, a direct comparison of these studies was challenging due to the lack of a uniform performance evaluation method for the models. As a result, we selected the most frequently used performance metric, which limited our comparison to just 6 out of the 15 papers.

Sleep monitoring mats detecting sleep apnea and sleep quality usually combined the information from body movement and the respiratory and heart rates. The six articles that were reviewed for sleep apnea included a wide range of patient populations, ranging from healthy to those with mild, moderate, and severe sleep apnea. Three of the studies used a sample size ranging from 31 to 136 subjects. Therefore, we can conclude that the detection accuracy of these studies was properly validated. From the five studies reviewed involving sleep quality, only one study included healthy subjects and sleep apnea subjects. Additionally, three of these studies had sample size of less than 10, indicating that the results are still in the preliminary stage and need further investigation. However, it should be noted that this is a new field of investigation and has only recently been examined, which explains the limited research completed.

Almost all the commercial sleep mats that provide heart rate, respiratory rate, and body movement data are not affordable, with the cheapest product being the Withings Sleep Tracking Mat at CAD 127.87. The Emfit monitoring system has the highest accuracy for estimating the respiratory rate and heart rate, with accuracies of 97.6% and 98.7%, respectively. However, this monitoring system requires a subscription of CAD 24.39/month, which may not be affordable for most people. The EarlySense system provides the highest accuracy rates for sleep cycle detection. However, this product is only available to medical institutions, long-term care homes, and rehabilitation centers. Therefore, although there are commercial products available for use in the market, they are either not affordable or require further validation to determine their efficacy.

## 5. Conclusions

This paper provides a review of smart mats that monitor body position and movement, physiological data, such as the heart rate and respiratory rate, sleep apnea, and sleep quality. Although all studies reviewed provided valuable solutions for sleep monitoring, reaching clear conclusions about the best physiological signals, sensor types, and algorithms for sleep monitoring has been challenging. This difficulty arises from the wide range of signal, sensor, and algorithm combinations used in the studies, as well as variations in participant health conditions and sample sizes. To provide more precise recommendations for a particular sleep monitoring sensor, future studies could focus on maintaining consistency in signal type, algorithm type, and participant characteristics while systematically alternating the sensor technology. Most of the papers reviewed for body posture monitoring were conducted in a simulated setting where the subjects were told which positions to lie in and for how long. However, this does not allow the accuracy or statistical data to be transferable to overnight environments or long-term studies, which is typically where the monitoring system would be used. Therefore, to ensure that body posture monitoring can be used in realistic settings, such as sleeping overnight, further research is required to evaluate the sensors and algorithms that are used in the studies we discussed. Though smart mats would be an affordable and accessible method for detecting heart and breathing rates during sleep, when studying the detection of physiological data and sleep quality, many of these papers did not include subjects that had cardiac, respiratory, or sleep disorders due to the low-quality signals. Therefore, in the future, studies should consider participants with similar characteristics as the target user population, such as those with cardiac or respiratory conditions and those with sleep disorders. The commercial products currently available on the market are not affordable enough for most people and require further validation. Therefore, research into more affordable sensors such as flexible transistor sensor arrays for sleep monitoring and appropriate validation in realistic use cases and target users is required. Although there has been a great deal of research conducted on smart mats, most of the smart mats used in these studies have not been available for practical use in either homes or hospitals.

## Figures and Tables

**Table 1:** Body posture classification studies with merged supine and prone positions or prone position excluded. The order is from highest to lowest detection accuracy rates.

Year	Ref.	#Subs	#Postures	Testing Condition	Type and Sensor No.	Algorithm	Accuracy
2021	[40]	13	3	Simulated	2048 pressure	CNN ^1^-to-SNN ^2^ conversion	100%
2023	[35]	13	3	Simulated	2048 pressure	ResNet-18	99.97%
						I3D ^3^	98.90%
2021	[36]	13	3	Simulated	2048 pressure	SVM ^4^	99.20%
						KNN ^5^	99.80%
						Deep NN ^6^	99.97%
2018	[31]	19	3	Clinical	784 pressure	Deep NN	99.70%
2016	[23]	10	5	Simulated	2048 FSR	HoG ^7^ and deep NN	98.10%
2020	[10]	3	5	Simulated	2 piezoelectric and 2 pressure	NN and BN ^8^	95.09%
2011	[24]	6	5	Simulated	2038 FSA	KNN	97.70%
2013	[25]	20	8	Simulated	2048 FSR	KNN	97.10%
2017	[38]	6	9	Simulated	6 FSR	Pattern matching	96.10%
2022	[43]	1	5	Clinical	1080 pressure and 1080 piezoelectric	LSTM ^9^	94.74%
2016	[37]	NA	3	Simulated	512 pressure	SVM	94.14%
2014	[26]	9	3	Simulated	1728 resistive	KNN	91.60%
2011	[27]	3	3	Simulated	1024 FSA	HMM ^10^	90.40%
2021	[28]	5	6	Simulated	1024 pressure	HoG and SVM	83.36%
						CNN	86.94%
2017	[44]	13	3	Simulated	2048 pressure	Deep NN	82.70%
2015	[29]	10	6	Simulated	1768 pressure	SVM	77.10%

CNN ^1^ = convolution neural network, SNN ^2^ = spiking neural network, I3D ^3^ = inflated 3D network, SVM ^4^ = support vector machine, KNN ^5^ = K-nearest neighbor, NN ^6^ = neural network, HoG ^7^ = histogram of oriented gradients, BN ^8^ = batch normalization, LSTM ^9^ = long short-term memory, and HMM ^10^ = hidden Markov model.

**Table 2:** Sleep posture classifications with supine and prone positions distinguished. The order is from highest to lowest detection accuracy rates.

Year	Ref.	#Subs	#Postures	Testing Condition	Type and Sensor No.	Algorithm	Accuracy
2020	[4]	12	4	Simulated	1728 piezoresistive	Artificial NN	97.9%
2019	[42]	300	6	Simulated	2048 piezoresistive	CNN	97.2%
2018	[31]	19	4	Clinical	784 pressure	Deep NN	97.1%
2021	[45]	7	4	Simulated	1024 pressure	SVM	95.54%
						KNN	96.43%
2022	[32]	16	4	Simulated	1024 FSR	FCSNet	95.43%
		5		Clinical		Tiny-MobileNetV2	86.80%
2021	[8]	16	4	Simulated	1024 FSR	ResNet	95.08%
		5		Clinical			86.35%
2010	[41]	3	9	Simulated	Video and FSR	SVM	94.05%
2016	[33]	14	6	Clinical	8192 piezoelectric pressure	EMD ^11^ and KNN	91.21%
2022	[46]	6	4	Simulated	330 pressure sensors	Sparse classifier	91.00%
2015	[47]	14	6	Simulated	8192 pressure	KNN	90.78%
2021	[11]	NA	6	Simulated	171 piezoresistive	CNN	90.5%
2021	[30]	11	5	Simulated	5 pressure tiles and 8 electrodes for capacitive sensing	Random forest	85.02%
2014	[34]	14	6	Clinical	8192 FSR	Sparse classifier	83.2%

EMD ^11^ = empirical mode decomposition.

**Table 3:** Studies for detecting the respiration rate. The order is from latest to oldest articles.

Year	Ref.	#Sub.	Testing Condition	Type and #Sensor	Algorithm	Coverage	Performance
2023	[55]	5	Simulated	13 sensing elements	Peak Detection	N/A	MAE ^12^: 0.65 bpm ^13^
2021	[53]	15	Simulated	2016 pressure sensors	DSST ^14^ shoulder blade	N/A	RMSE ^15^: 1.32 bpm
					DSST with weighted centroid	N/A	RMSE: 0.87 bpm
2021	[56]	10	Clinical	Plastic optical fiber	Peak detection	N/A	RE ^16^: 6.7%
2020	[12]	10	Clinical	Microbend fiber optic sensor	Wavelet analysis and EMD	N/A	NMAE ^17^: 11.42% ± 2.62
2019	[14]	10	Simulated	18 piezoelectric ceramic sensors	Wavelet analysis	N/A	ACC ^18^: 98.95%
					EMD		ACC: 95.12%
					DS		ACC: 96.06%
2019	[59]	80	Clinical	Microbend FOS ^19^	MODWT	76.62%	MAE: 4.57 ± 6.89 bpm
					HNM ^20^	76.62%	MAE: 6.29 ± 8.14 bpm
					CLIE ^21^	76.62%	MAE: 8.71 ± 8.37 bpm
2018	[50]	10	Simulated	FOS sensors	Peak detection	N/A	MAE: 0.38 ± 0.32 bpm
2016	[61]	4	Clinical	4 load cells	Peak detection	84.25%	MAE: 2.66%
2015	[58]	5	Clinical	5 load cells	Peak detection	N/A	MAE: 0.18 bpm
2012	[60]	6	Clinical	Emfit sensors and 8PVDF ^22^	PCA model ^23^	90.00%	MAE: 1.5%

MAE ^12^ = mean absolute error, bpm ^13^ = breaths per minute, DSST ^14^ = de-shape synchrosqueezing transform, RMSE ^15^ = root mean squared error, RE ^16^ = relative error, NMAE ^17^ = normalized mean absolute error, ACC ^18^ = accuracy, FOS ^19^ = fiber optic sensor, HNM ^20^ = harmonic plus noise model, CLIE ^21^ = continuous local interval estimation, PVDF ^22^ = polyvinylidene fluoride, and PCA model ^23^ = principal component analysis model.

**Table 4:** Heart rate studies’ details. The order is from latest to oldest articles.

Year	Ref.	#Sub.	Testing Condition	Type and #Sensor	Algorithm	Coverage	Performance
2021	[53]	15	Simulated	2016 pressure sensors	DSST	N/A	RMSE ^24^: 5.55 bpm
2021	[54]	10	Simulated	4 fiber Bragg grating sensor arrays	Template matching	N/A	MAE: 3.31 ± 1.26 bpm
2021	[56]	10	Clinical	Plastic optical fiber	Peak detection	N/A	RE: 2.4%
2020	[12]	10	Clinical	Microbend fiber optic	Wavelet analysis and EMD	N/A	NMAE: 5.42% ± 0.57
2019	[14]	10	Simulated	18 piezoelectric ceramic	Wavelet analysis	N/A	ACC: 98.11%
					EMD		ACC: 96.19%
					DS		ACC: 95.08%
2018	[51]	6	Simulated	FOS	Cepstrum	N/A	MAE: 4.62 ± 1.68 bpm
					MODWT		MAE: 6.87 ± 1.94 bpm
					CEEMDAN		MAE: 7.85 ± 4.34 bpm
					Clustering		MAE: 2.76 ± 9.53 bpm
2018	[50]	10	Simulated	FOS	MODWT	N/A	MAE: 0.55 ± 0.59 bpm
2016	[61]	4	Clinical	4 load cells	Peak detection	73.79%	MAE: 2.55%
2015	[15]	2	Simulated Clinical	Capacitive sensors	R-peak detection	98.70%	MNE ^25^: 0.28%
		7				97.80%	MNE: 0.15%
2012	[60]	28	Clinical	Emfit sensors and 8 PVDF	Cepstrum	80.00%	MAE: 1.8%
2012	[57]	4	Clinical	8 embroidered textile electrodes	R-peak detection	94.90%	RMSE: 0.27 bpm
2012	[52]	4	Simulated	Hydraulic bed sensor	k-means clustering	97.90%	N/A

bpm ^24^ = beats per minute and MNE ^25^ = mean normalized error.

**Table 5:** Sleep apnea studies. The order is from latest to oldest articles.

Year	Ref.	#Sub.	Testing Condition	Signal Used	Type of Sensor	Algorithm	Performance
2020	[12]	10	Clinical	RR ^26^	Microbend fiber optic sensor	Wavelet analysis and EMD	ACC: 54.33%
2018	[50]	10	Simulated	RR, HR ^27^, BM ^28^	FOS	Peak detection and MODWT	Sen ^29^: 24.24% Spec ^30^: 85.88%
2017	[20]	136	Clinical	RR, HR	Micro-movement-sensitive mattress	Peak detection	ACC: 93.2%
						KNN	ACC: 95.05%
						SVM	ACC: 93.02%
						Random forest	ACC: 97.57%
2017	[65]	9	Clinical	RR, BM	Strain gauges	Naive Bayes	ACC: 71.9%
2016	[69]	96	Clinical	RR, HR, BM	Piezoelectric	Peak detection	Sen: 88%
					sensors		Spec: 89%
2014	[63]	31	Clinical	RR, BM	PVDF-based sensors	PCA	ACC: 86.68%

RR ^26^ = respiratory rate, HR ^27^ = heart rate, BM ^28^ = body movement, Sen ^29^ = sensitivity, and Spec ^30^ = specificity.

**Table 6:** Sleep quality studies. The order is from latest to oldest articles.

Year	Ref.	#Sub.	Testing Condition	Data	Type of Sensor	Algorithm	Performance
2020	[67]	5	Clinical	RR, BM	Piezoresistive sensors	ANN	ACC: 86%
2019	[70]	25	Clinical	BM	4 pressure sensors	HMM	ACC: 90.9%
2014	[13]	7	Clinical	RR, BM	Piezoresistive sensors	KNN	ACC: 67.12%
						SVM	ACC: 70.33%
						NB ^31^	ACC: 72.2%
2014	[66]	20	Clinical	HR, BM	PVDF film sensor	Peak detection	ACC: 96.95%
2010	[68]	9	Clinical	HR, BM	Emfit foil electrodes	HMM	ACC: 78.67%

NB ^31^ = naïve Bayes.

**Table 7:** A summary of all validation studies completed on the described commercial products.

Year	Ref.	#Sub.	Testing Condition	Signal Monitored	Sleep Mat	Performance
2021	[19]	118	Clinical	Sleep apnea detection	Withings mat	Average ACC: 65.09%
2019	[9]	24	Clinical	RR	Withings mat	ACC: 69.6%
		37		HR		ACC: 40%
2019	[72]	10	Clinical	Sleep cycle	Beddit mat	Average ACC: 45.07%
2019	[75]	20	Clinical	HR	Emfit mat	RMSE: 2.4 bpm
2019	[76]	34	Clinical	HR	Emfit mat	ACC: 98.7%
				RR		ACC: 97.6%
2018	[81]	21	Clinical	RR	Studio 1 Labs mat	ACC: 84%
2017	[79]	63	Clinical	HR	EarlySense	ACC: 96.1%
				RR		ACC: 93.3%
2017	[80]	28	Clinical	Sleep cycle	EarlySense	Average ACC: 72.1%

## Data Availability

Not applicable.
